# Characterization of a Protein Phosphatase Type-1 and a Kinase Anchoring Protein in *Plasmodium falciparum*

**DOI:** 10.3389/fmicb.2018.02617

**Published:** 2018-10-31

**Authors:** Astrid Lenne, Caroline De Witte, Géraldine Tellier, Thomas Hollin, El Moukhtar Aliouat, Alain Martoriati, Katia Cailliau, Jean-Michel Saliou, Jamal Khalife, Christine Pierrot

**Affiliations:** ^1^INSERM U1019-CNRS UMR 8204, Center for Infection and Immunity of Lille, Institut Pasteur de Lille, Université de Lille, Lille, France; ^2^CNRS, INRA, UMR 8576-Unité de Glycobiologie Structurale et Fonctionnelle, Université de Lille, Lille, France

**Keywords:** *Plasmodium*, protein phosphatase type-1, regulator of chromosome condensation, CDPK7, protein-protein interaction

## Abstract

With its multiple regulatory partners, the conserved Protein Phosphatase type-1 (PP1) plays a central role in many functions of the biology of eukaryotic cells, including *Plasmodium falciparum*. Here, we characterized a protein named PfRCC-PIP, as a major partner of PfPP1. We established its direct interaction *in vitro* and its presence in complex with PfPP1 in the parasite. The use of *Xenopus* oocyte model revealed that RCC-PIP can interact with the endogenous PP1 and act in synergy with suboptimal doses of progesterone to trigger oocyte maturation, suggesting a regulatory effect on PP1. Reverse genetic studies suggested an essential role for RCC-PIP since no viable knock-out parasites could be obtained. Further, we demonstrated the capacity of protein region containing RCC1 motifs to interact with the parasite kinase CDPK7. These data suggest that this protein is both a kinase and a phosphatase anchoring protein that could provide a platform to regulate phosphorylation/dephosphorylation processes.

## Introduction

Malaria is a severe parasitic infection and remains a leading cause in morbidity and mortality among children, particularly in sub-Saharan Africa. The lack of an effective vaccine and the emergence of artemisinin resistant-parasites could contribute to worsen the overall health and socio-economic situation in endemic areas. Therefore, it is important to continue to gain a better understanding of the biology of *Plasmodium* in order to open up new strategies for disease intervention. In this context, it has been reported that several kinases and phosphatases, catalyzing protein phosphorylation and dephosphorylation events, respectively, are vital in the development of malaria parasites (Doerig et al., 2015). Indeed, reverse genetic approaches in both *Plasmodium falciparum* and *Plasmodium berghei* suggested that most kinases and phosphatases could be essential for the completion of parasite life cycle (Tewari et al., 2010; Solyakov et al., 2011; Guttery et al., 2014). Protein Phosphatase type-1 (PP1), one of the main catalytic and conserved subunits known to dephosphorylate serine and threonine residues, has emerged as an indispensable enzyme for the growth and differentiation of blood stage parasites (Tewari et al., 2010; Zhang et al., 2012).

Several studies demonstrated that yeast and mammalian PP1 is a key regulatory actor in diverse cellular function including the control of gene transcription, protein synthesis and cell division (Cohen, 2002; Rebelo et al., 2015). To explain these multiple roles, there is growing evidence showing that regulatory subunits, grouped more commonly as PP1 interacting proteins (PIPs), are required to successfully fine tune and to adapt PP1 targeting, specificity and activity. So far, 189 proteins have been shown to directly interact with PP1 and to participate in its regulatory code (Hendrickx et al., 2009; Fardilha et al., 2010; Heroes et al., 2013). These PIPs could be functionally classified in three groups. The first is constituted by regulators of PP1 activity, the second includes targeting proteins contributing to direct PP1 toward specific subcellular locations and the third group is composed of PP1 substrates, which could also encompass the first two groups (Bollen, 2001). Although most of these interactors exhibit no significant amino acid sequence similarities, ruling out any structural classification, 85% of PIPS (162/189) share one main binding motif corresponding to the RVXF consensus sequence where X represents any amino acid except proline (Choy et al., 2014). Further studies combining sequence alignments, deletions and point mutations has refined this binding motif as [RK]-X_0-1_[VI]-{P}-[FW] where X denotes any residue and {P} any residue except proline (Zhao and Lee, 1997; Wakula et al., 2003).

In *P. falciparum* (Pf), our initial studies based on sequence alignments between well-known regulators and putative Pf proteins led to the identification of PfLRR1 (an ortholog of yeast or human Sds22), Pf Inhibitor-2 (PfI2), Pf Inhibitor-3 (PfI3), and PfeIF2ß (Daher et al., 2006; Freville et al., 2012, 2013; Tellier et al., 2016). Structure-interaction studies revealed that the interaction of PP1-PfLRR1 involved one LRR and the LRR cap motif (Pierrot et al., 2018) while PfI2, PfI3, and PfeIF2ß have been shown to interact with PfPP1 via their RVXF motifs. Functional studies indicated that three of these interactors were able to regulate the phosphatase activity of PfPP1. With regard to the function of PfeIF2ß, we observed a divergence with its human counterpart since the former did not affect PP1 activity while the latter has been shown to be a potent inhibitor (Wakula et al., 2006). Reverse genetic studies in Pf suggested the essentiality of these PIPs for blood stage parasites (Freville et al., 2012, 2013; Tellier et al., 2016). Interestingly, synthetic peptides derived from PIPs binding motifs capable of disrupting the binding of the corresponding PIPs to PfPP1 were able to inhibit parasite growth *in vitro*, underscoring the importance of these interactions for the completion of the parasite intra-erythrocytic life cycle (Freville et al., 2013, 2014; Pierrot et al., 2018). These observations clearly underline the importance of the identification of novel PIPs in Pf. In this context, *in silico* screening of Pf genes containing an extended and refined RVXF sequence, together with experimental approaches including yeast two-hybrid (Y2H) screening in which PfPP1 was used as bait, allowed us to describe the first PfPP1 interactome (Hollin et al., 2016). In this earlier work, eight clones (4% of the clones sequenced) revealed by Y2H screening under stringent conditions were found to correspond to the same region of a protein annotated as putative Regulator of Chromosome Condensation (RCC) protein (PF3D7_0919900) (Aurrecoechea et al., 2009; Ochoa et al., 2011). This annotation was based on the presence of RCC1 repeats predicted using the InterPro Database (Mulder et al., 2005). Interestingly, this gene was also detected via the *in silico* approach (Hollin et al., 2016), and further analysis of the deduced amino acid sequence from the Y2H clones confirmed a shared potential interacting region containing the RVXF motif. Altogether, these data support the participation of PF3D7_0919900 in the PP1 network.

In the present study, PF3D7_0919900 was further characterized at the molecular and functional levels. We established by additional approaches that it is a direct interactor of PfPP1, and showed the role of the RVXF motif in this interaction. Expression of the PP1-interacting region of PF3D7_0919900 in *Xenopus* oocytes model revealed that it is functional. Studies in the parasite showed that PF3D7_0919900 is cytoplasmic and interacts *in situ* with PfPP1. Reverse genetics strongly suggested an essential role of this protein since no viable knock-out (KO) parasites could be obtained. Finally, since PF3D7_0919900 contains RCC motifs known to be involved in protein-protein interactions, we undertook Y2H screening using the RCC region of PF3D7_0919900 as bait and showed that it interacts with the parasite kinase CDPK7. Based on these data, we designate this gene as PfRCC-PIP for *P. falciparum* Regulator of Chromosome Condensation-PP1-Interacting Protein and suggest that it may be involved in the regulation of both phosphorylation and dephosphorylation processes.

## Materials and Methods

### Plasmids

Plasmids pCR^TM^2.1-TOPO^®^, pQE30, pGEX4T3, pGADT7, and pGBKT7 were purchased from Invitrogen, Qiagen, Life Sciences and Clontech, respectively. Plasmids used in reverse genetic studies in *P. falciparum*, pCAM-BSD and pCAM-BSD-HA, were kind gifts from Prof. C. Doerig (Monash University, Melbourne, VIC, Australia). Plasmids used in *P. berghei* reverse genetic studies, p-TRAD4Ty-TetO7-HA-hDHFR and pBS-DHFR, were given by Prof. D. Soldati-Favre (University of Geneva, Switzerland) and Prof. R. Tewari (University of Nottingham, United Kingdom), respectively.

All primers used in this study are indicated in Supplementary Table 1.

### Overlapping PCR

The coding sequence of PfRCC-PIP was checked by overlapping PCR performed on first strand cDNA prepared from *P. falciparum* total RNA. RNA was treated with DNase I (Invitrogen) before the reverse transcription reaction, and the absence of contamination by genomic DNA was verified using primers of an intron-containing unrelated gene. Overlapping PCRs were carried out using Advantage 2 PCR kit (Clontech) and ten pairs of primers (p1 to p20, see Supplementary Table 1) designed according to the predicted sequence of PfRCC-PIP (PF3D7_0919900).

### 3D Modeling

The modeling of the RCC motifs of PF3D7_0919900 (AA 140 to 424), based on the human RCC1 protein (BAA00469.1), was carried out via the modbase website^1^.

### Parasites Cultures

The 3D7 clone of *P. falciparum* was grown as previously described (Freville et al., 2012). To obtain ring stage and carry out transfections, parasites were synchronized by a double sorbitol treatment as previously described (Vernes et al., 1984). The genomic DNA (gDNA) was extracted as previously described (Tellier et al., 2016).

### Generation and Genotyping of *P. falciparum* Transgenic Parasites

In order to tag the endogenous PfRCC-PIP, a portion of the 3′ end of PF3D7_0919900 (710 bp omitting the stop codon) was amplified with primers p25-p26 (Supplementary Table 1) and cloned in the pCAM-BSD-HA plasmid. This plasmid contains a cassette conferring resistance to BSD. Transfections, parasites culture and selection of BSD-resistant parasites were carried out as previously described (Tellier et al., 2016). The integration of the construct was checked by PCR on gDNA with primers p29-p28 (Supplementary Table 1), and episomal DNA was detected with primers p27-p28.

A knock-out was carried out using pCAM-BSD as disruption plasmid. A 5′ fragment of PF3D7_0919900 (847 bp) amplified with p51-p52 was cloned in this plasmid. The genotype of BSD-resistant parasites was analyzed by PCR on gDNA using primers p55-p54 to detect an integration, and p53-p54 to detect episomal DNA (Supplementary Table 1).

### Generation and Genotyping of *P. berghei* Transgenic Parasites

To replace the PbRCC-PIP gene by double homologous recombination, a pBS-DHFR vector was used (Guttery et al., 2012). Fragments corresponding to the 5′ upstream and 3′ downstream sequence of the PbRCC-PIP gene (PBANKA_0820800) were amplified using primers p56-p57 and p58-p59, respectively, and inserted in the pBS-DHFR vector. The construct was linearized before transfection as described (Guttery et al., 2012).

For HA-tagging of PbPP1, promoter and coding regions of PBANKA_1028300, obtained using primers p38-p39 and p40-p41, respectively, were cloned into the p-TRAD4Ty-TetO7-HA-hDHFR plasmid (Pino et al., 2012). Before transfection, the sequence was linearized using *Bg*lII.

*P. berghei* ANKA transfections were performed by electroporation of schizont stages according to Janse et al. (2006), using 10 μg of linearized constructs for each transfection. The schizont stages were obtained from 6 week-old infected Fisher rats (Charles River) and separated on a 55% Nycodenz gradient after 16 h culture at 37°C in RPMI1640 culture medium supplemented with 0.4% AlbuMAX^TM^ II Lipid-Rich BSA (Life Technologies).

Genotyping was performed in parallel on total DNA from parental and transfected parasites extracted from schizont pellets using the KAPA Express Extract Kit (KAPABioSystem). Primers used for genotyping are indicated in Supplementary Figures 2, 4 and Table 1.

**Table 1:** Detection of PbRCC-PIP in complex with PbPP1-HA after IP/MS analysis.

Protein name	PlasmoDB	Total number of				Total number of			
	accession number	unique peptides				spectra			
		Expt 1		Expt 2		Expt 1		Expt 2	
		WT^a^	PP1^b^	WT^a^	PP1^b^	WT^a^	PP1^b^	WT^a^	PP1^b^
Serine/threonine protein phosphatase (PbPP1)	PBANKA_1028300	4	25	0	19	5	288	0	152
Leucine-rich repeat protein (PbLRR1)	PBANKA_0516600	0	19	0	16	0	107	0	46
Protein phosphatase inhibitor 2 (PbI2)	PBANKA_1218500	0	8	0	8	0	54	0	36
Putative regulator of chromosome condensation (PbRCC-PIP)	PBANKA_0820800	0	24	0	14	0	35	0	26

*^*a*^Immunoprecipitation performed with extracts from parental parasites.*

*^*b*^Immunoprecipitation performed with extracts from HA-tagged PbPP1 parasites.*

### Immunofluorescence Assay

An asynchronous culture of *P. falciparum* parasites expressing HA-tagged PfRCC-PIP (5% parasitemia) was centrifuged and fixed (paraformaldehyde 4% and glutaraldehyde 0.075%) 10 min on ice. After centrifugation, erythrocytes were suspended in PBS and incubated in 24-well tissue culture plate containing poly-L-lysine-coated coverslips for 30 min at room temperature. Coverslips were then washed with PBS and permeabilization of red blood cells was performed in PBS BSA 1%, Triton X-100 0.5% for 3 min at room temperature. Coverslips were washed and incubated with biotinylated anti-HA antibodies (Roche, 1:100) 1 h at 37°C in a humid chamber. After additional washings, coverslips were incubated with streptavidin-AF488 (Molecular Probes) diluted 1:200 in 1% BSA PBS and 1 μg/ml DAPI (Sigma-Aldrich), for 1 h at 37°C in a humid chamber in the dark. The conjugate control consists of erythrocytes solely incubated with streptavidin-AF488. The coverslips were washed in PBS before mounting the slide in Mowiol 4-88 (Sigma-Aldrich). The slides were then observed in confocal microscopy by the LSM880 microscope (Zeiss).

### Directed Mutagenesis

Site-directed mutagenesis experiments were performed on the RVXF binding motif of PfPP1 (^255^FF^256^/AA) as well as on the RVXF motif of the PIP region of PfRCC-PIP (^980^KSASA^984^) using primers p21-p22 and p23-p24, respectively, and wild type constructs as templates. Isis Proofreading DNA Polymerase (MP Biomedicals) was used following the manufacturer’s recommendations. The parental DNA plasmid was digested with *Dpn*I (Life Technologies) and an aliquot was used to transform XL10-Gold Ultracompetent cells (Agilent) for pGADT7 construction, or M15 cells for pQE30 construction. Mutants were checked by sequencing and used in yeast two-hybrid system or in recombinant proteins expression.

### Interaction Studies and Screening in Yeast Two-Hybrid System

The interaction between PfRCC-PIP and its partners was carried out in yeast two-hybrid system as previously described (Freville et al., 2013). HA-tagged proteins were extracted using Yeast Protein extraction buffer kit (GE Healthcare) as described in the manufacturer’s protocol and the presence of the protein was observed by western blot with anti-HA antibodies (Roche, 1:2,500) and horseradish peroxidase-labeled anti-mouse IgG (Santa Cruz; 1:50,000). The production of cMyc-labeled proteins in yeast was checked on an overnight culture. The culture was centrifuged 5 min at 1,000 g, and the pellet was suspended in 100 μl of water. One hundred micro liter of NaOH 0.2 M were then added and the solution was incubated 5 min at room temperature. The lysate was centrifuged 5 min at 1,000 g. The pellet was suspended in 100 μl of Laemmli buffer, heated 3 min at 100°C, and proteins were separated by electrophoresis. The cMyc-tagged proteins were detected by western blot using an anti-cMyc antibody (Sigma-Aldrich; 1:1,000) and HRP-labeled anti-mouse IgG (1:50,000).

To determine the interactome of RCC motifs, the RCC region of PfRCC-PIP (AA 1 to 399) was obtained using primers p45 and p46, and cloned in pGBKT7 vector using the In-fusion HD cloning Kit (Clontech). To examine interaction partners of RCC region, a yeast two-hybrid screen was performed as previously described (Hollin et al., 2016).

### Recombinant Protein Expression and GST Pull-Down Assays

The wild type and ^980^KSASA^984^-mutated PIP region of RCC-PIP were produced as His-tagged recombinant proteins in *E. coli* as described previously (Hollin et al., 2016) with the following modifications: the induction of the expression was carried out overnight at 16°C and 150 mM NaCl was used in sonication, washing and elution buffers. The recombinant protein corresponding to the His-tagged RCC region of PfRCC-PIP was produced as described (Hollin et al., 2016). All His-tagged recombinant proteins were purified according to manufacturer’s instructions by Ni^2+^ chelation chromatography (GE Healthcare). The purity of each protein, checked by SDS–PAGE followed by SimplyBlue^TM^ safe staining (Invitrogen) was >90%. Quantification of recombinant proteins was performed using the Pierce^TM^ BCA Protein Assay Kit (Life Technologies) following the manufacturer’s instructions.

PfPP1-GST, GST alone, and CDPK7-GST (cloned with primers p49-p50 in pGEX4T3) recombinant proteins were produced in *E. coli* as previously described (Tellier et al., 2016) and were coupled to glutathione agarose (Sigma-Aldrich). Two μg of the RCC region of PfRCC-PIP-6His recombinant protein, wild type or ^980^KSASA^984^-mutated, were incubated with GST alone, PfPP1-GST or PfCDPK7-GST bound to glutathione agarose beads, BSA and binding buffer as previously described (Tellier et al., 2016).

### Antisera Production

For antisera production, the His-tagged recombinant protein corresponding to the PIP region of PfRCC-PIP (AA 863–1108, predicted MW 28.8 kDa) was, mixed with Alu-Gel-S (Serva) (1v/1v) and injected i.p. into 8–10 week-old male CD1 mice (Charles River). The pre-immune sera were collected before injection and used as a negative control. Animals were boosted twice at days 21 and 35 post injection under the same conditions. The sera were collected at day 49, and then every 2 weeks until a decrease in titer was observed by western blot.

### IP/MS Experiments

Immunoprecipitation of HA-tagged PbPP1 was carried out on schizonts obtained by a 50% Nycodenz gradient purification. Parental parasites were used as control. Pellets of schizonts were suspended in lysis buffer [Tris–HCl 50 mM, Triton X100 0.5% and protease inhibitor cocktail (Roche), pH 8]. The samples were treated with ten freeze-thawing cycles (liquid nitrogen/water bath 37°C), sonicated and centrifuged for 1 h at 13,000 rpm at 4°C, and the supernatants were used as soluble fractions. These fractions were incubated overnight on a rotation wheel at 4°C with 100 μl anti-HA agarose beads (Life Technologies) pre-saturated with BSA in washing buffer (Tris 20 mM, NaCl 150 mM, Triton X100 0.5%, inhibitor protease cocktail, pH 7,5). Beads were washed ten times and PbPP1-associated proteins were eluted twice in 15 μl of Laemmli buffer. After 3 min at 100°C, samples were loaded on a 4–20% SDS–PAGE for mass spectrometry analysis. Electrophoretic migration, tryptic digestion and nano LC-MSMS analysis were performed as previously described (Lesage et al., 2018). Raw data collected during nano LC-MS/MS analyses were processed and converted into ^∗^.mgf peak list format with Proteome Discoverer 1.4 (Thermo Fisher Scientific). MS/MS data were interpreted using search engine Mascot (version 2.4.0, Matrix Science, London, United Kingdom) installed on a local server. Searches were performed with a tolerance on mass measurement of 0.2 Da for precursor and 0.2 Da for fragment ions, against a composite target decoy database (2^∗^21,948 total entries) built with *Mus musculus* UniProt database (10,090–16,754 entries), *P. berghei* PlasmoDB database (March 2017 – 5,112 entries) fused with the sequences of recombinant trypsin and a list of classical contaminants (46 entries). Cysteine carbamidomethylation, methionine oxidation, protein N-terminal acetylation, and cysteine propionamidation were searched as variable modifications. Up to one trypsin missed cleavage was allowed. For each sample, peptides were filtered out according to the cut-off set for proteins hits with peptides taller than nine residues, ion score >40, identity score >5, and a false positive rate of 1%.

### Experiments in *Xenopus laevis* Oocytes

cRNA encoding wild type or ^980^KSASA^984^-mutated PIP region of RCC-PIP were transcribed using the T7 mMessage mMachine^®^ Kit (Ambion) and from the T7 promotor-containing pGADT7 plasmids (1 μg). These plasmids were previously linearized with *Hind*III. cRNA were produced according to the manufacturer’s recommendations.

Preparation of *Xenopus* oocytes and micro-injection experiments were performed as previously described (Vicogne et al., 2004). In each assay, 20 oocytes from 2 or 3 different animals were micro-injected with 60 ng of cRNA coding for the wild type or ^980^KSASA^984^-mutated PIP region of PfRCC-PIP. Germinal Vesicle BreakDown (GVBD) was detected by the appearance of a white spot on the animal pole. Progesterone (PG) was used as positive control (10 μM). Oocyte extracts were prepared 15 h post micro-injection as previously described (Freville et al., 2013), and western blot was performed after SDS–PAGE to detect the presence of the wild type or ^980^KSASA^984^-mutated HA-tagged PIP region of PfRCC-PIP, using anti-HA antibodies (Invitrogen, 1:1,500). The interaction between the wild type or ^980^KSASA^984^-mutated PIP region of PfRCC-PIP and XePP1 was investigated by co-immunoprecipitation using anti XePP1 antibodies (Santa-Cruz Biotechnology) in the presence of Sepharose-protein G as described previously (Freville et al., 2013). Eluted fractions were analyzed by western blot using anti-XePP1 (Santa-Cruz Biotechnology, 1:1,000) or anti-HA antibodies (Invitrogen, 1:1,500), followed by horseradish peroxidase labeled anti-mouse or anti-rabbit secondary antibodies (Invitrogen, 1:30,000) and revealed by chemiluminescence detection (Amersham ECL select).

The effect on GVBD of the micro-injection of the cRNA encoding wild type or ^980^KSASA^984^-mutated PIP region of RCC-PIP was tested in the presence of increasing concentrations of PG (0.1 nM–10 μM). In kinetic assays, the GVBD appearance and the protein expression and interaction were assessed every hour from 1 to 10 h and then at 15, 24, and 48 h post micro injection.

### Ethics Statement

Animal studies were approved and supervised by the local Animal Ethics Committee in accordance with the French national regulations. The ethical approval number is 00527.04. The full name of the Ethics Committee is: C2EA-75 Comité d’Ethique en Expérimentation Animale Nord – Pas de Calais-France.

### Statistical Analysis

The Mann-Whitney U test for non-parametric data was used for statistical comparisons of percentages of GVBD observed in *Xenopus* oocytes. *P* < 0.05 was considered significant.

## Results

### Analysis and Functional Annotation of PF3D7_0919900

**FIGURE 1:**
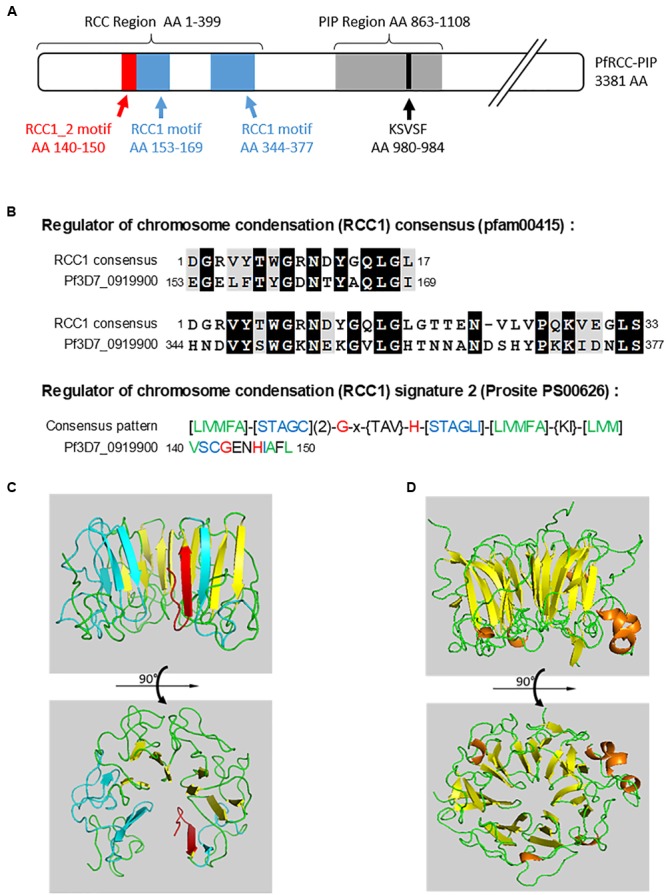
Sequence analysis of PfRCC-PIP. **(A)** Schematic representation of PfRCC-PIP. The RCC and PIP regions with the RVXF motif (in black), two RCC1 (in blue), and one RCC1_2 (in red) motifs are indicated. The portion of PfRCC-PIP detected by Y2H is highlighted in gray. **(B)** Alignments of the two RCC1 motifs of PfRCC-PIP with RCC1 consensus motif (pfam00415) and RCC1_2 of PfRCC-PIP with RCC1 signature 2 consensus motif (Prosite PS00626). **(C)** Predicted model of the portion of PfRCC-PIP containing the RCC1 motifs (AA 140–424), based on the structure of human RCC1 **(D)** (BAA00469.1) (see text footnote 1). Beta sheets are represented by arrows, RCC1 motifs are colored in blue and RCC1_2 motif in red.

In order to confirm the expression and the sequence of PF3D7_0919900 (assigned here as PfRCC-PIP), we performed overlapping PCRs on reverse transcribed Pf3D7 total RNA owing to the difficulty of obtaining the full length open reading frame of the gene (10146 bp, and 78% AT content). Using several primers pairs derived from the predicted sequence (Supplementary Table 1), 10 fragments of 963 to 1330 bp length were obtained and cloned in TA vector (Supplementary Figure 1). The sequencing of three clones for each fragment confirmed the predicted available sequence except for a single silent nucleotide mutation (T→C) at position 467. Analysis of the deduced amino acid (AA) sequence (3381 AA) confirmed the presence of the RVXF motif (^980^KSVSF^984^) observed in the clones of the Y2H screens (Supplementary Figure 1 and Figure 1A).

Further analysis of the sequence confirmed the presence of two Regulator of Chromosome Condensation (RCC1) consensus motifs (pfam00415) (AA 153–169 and 344–377) and one RCC1 signature 2 (AA 140–150) (Prosite PS00626) (Figures 1A,B) in the Nt region of PfRCC-PIP. These motifs are present in RCC1-like domains (RLD), a feature of RCC1 superfamily proteins (Hadjebi et al., 2008). These motifs, which can vary from three to seven in copy number are known to be involved in interactions with proteins and chromatin. RCC1 motifs of PfRCC-PIP show conserved glycines and hydrophobic residues, well described to provide the characteristic seven-bladed propeller structure of RLD-containing proteins (Renault et al., 1998). This prompted us to build a 3D model of the RCC1 motif-containing region of PfRCC-PIP using human RCC1 (BAA00469.1) as a template (see text footnote 1). The overall structure of this region is composed of 14 antiparallel ß sheets in which the first 2 and the last 2 correspond to RCC1 motifs (Figure 1C). While being different from the characteristic seven-blades structure of RCC1 superfamily proteins, this 3D model shows an overall conserved structure that could allow the binding of proteins and/or DNA (Figures 1C,D).

Syntenic orthologs of PfRCC-PIP are present in *Plasmodium spp*. This concerns particularly the *P. berghei* gene PBANKA_0820800 whose deduced amino acid sequence (2518 AA) shows an overall identity of 28% with PfRCC-PIP (data not shown). Three regions corresponding to AA 1–470, 862–1070, and 2280–2540 (AA positions in PfRCC-PIP) are highly conserved with 48, 31, and 46% identity, respectively. The first two regions include the RCC1 and RVXF motifs, respectively, that are conserved in the *P. berghei* gene.

### Targeted Gene Disruption of the RCC-PIP Gene

**FIGURE 2:**
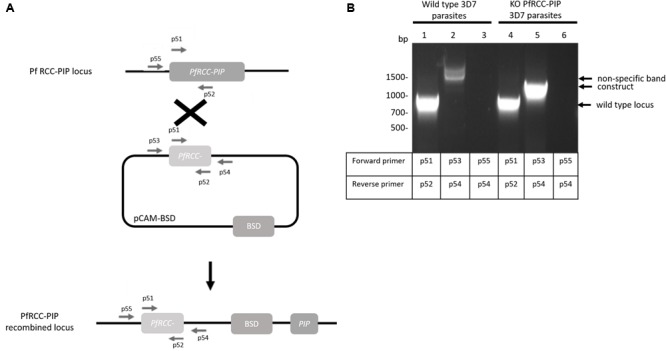
Targeted gene disruption of the PfRCC-PIP locus in *P. falciparum*. **(A)** Disruption of PfRCC-PIP by a knock-out strategy involving single homologous recombination using the pCAM-BSD vector. The pCAM-BSD construct, the blasticidin-resistance cassette (BSD), the location of the primers used for PCR analysis and the locus resulting from integration are shown. **(B)** Diagnostic PCR analysis of pCAM-BSD-PfRCC-PIP-transfected 3D7 cultures; lanes 1 to 3 correspond to DNA extracted from wild type 3D7 parasites and lanes 4 to 6 to DNA extracted from transfected parasites. Lanes 1 and 4 represent the detection of the wild type locus (PCR with p51-p52); lanes 2 (presence of non-specific band) and 5 (presence of the specific band at the expected size) represent the detection of the construct (PCR with p53-p54) and lanes 3 and 6 correspond to the integration at the 5′ end of the insert (PCR with p55-p54).

To investigate the functional role of RCC-PIP in *P. falciparum* (Pf), a knock-out approach using the pCAM vector system was undertaken (Figure 2A). Blood ring stage parasites were transfected with a pCAM-BSD-PfRCC-PIP vector containing a 5′ fragment derived from the PfRCC-PIP gene. From 3 independent transfection experiments, the analysis of genomic DNA by diagnostic PCR with specific primers, even after long period of culture (>10 months) with several BSD on/off cycles (Figure 2A and Supplementary Table 1), did not show the integration of the BSD resistance gene, indicating the absence of viable knock-out parasites (Figure 2B). The endogenous RCC-PIP gene was amplified in genomic DNA and the plasmid remained episomal (Figure 2B, lane 5). The absence of viable knock-out parasites could not be attributed to a lack of accessibility to the locus as a HA tag was accomplished by genetic knock-in (see below), suggesting that PfRCC-PIP could be essential to the Pf blood stage lifecycle. Next, we tried to delete the RCC-PIP homolog in *P. berghei* (Pb) since it has higher transfection efficiency than *P. falciparum*. The targeting vector was designed to replace the PbRCC-PIP gene with a resistance marker by double homologous recombination (Supplementary Figure 2). In four independent transfections, we failed to knock-out *PbRCC-PIP*. In these assays, transfections with a control construct were performed and resistant Pb parasites were obtained, excluding any technical issues.

### Interaction of the PIP Region of PfRCC-PIP With PfPP1 and Contribution of the RVXF Motif

**FIGURE 3:**
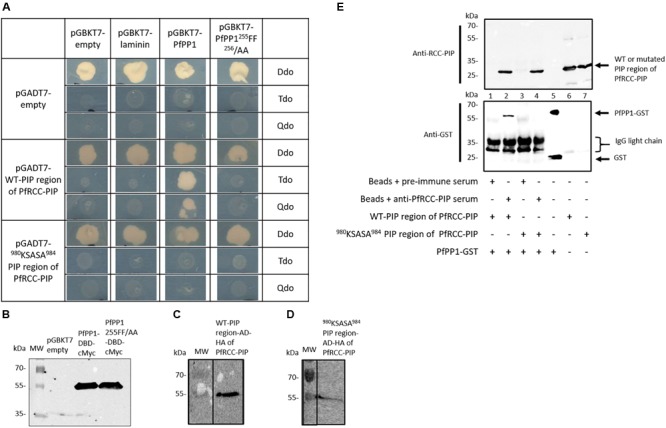
Direct interaction between the PIP region of PfRCC-PIP and PfPP1 and involvement of the RVXF motif. **(A)** Interaction in a yeast two-hybrid assay between the PIP region of PfRCC-PIP wild type (WT) and mutated (^980^KSASA^984^), with PfPP1 wild type and mutated (^255^FF^256^/AA). Constructs in pGBKT7 were inserted into mat α yeast (Y187 strain), pGADT7 empty and pGADT7-RCC-PIP were inserted in mat a yeast (Y2HGold strain). After transformation, mating experiments were carried out. Diploids were checked on Ddo medium, interactions and strong interactions were examined on Tdo and Qdo medium, respectively. The expression of proteins was checked by western blot analysis of extracts prepared from each yeast strain and revealed with anti-cMyc mAb for wild type and mutated (^255^FF^256^/AA) PfPP1 **(B)** and anti-HA mAb for wild type **(C)** and mutated **(D)** PIP region of PfRCC-PIP. **(E)** Interaction between PfPP1 and PIP region of PfRCC-PIP analyzed by immunoprecipitation. Beads coupled to antibodies raised against PfRCC-PIP or to control antibodies from pre-immune serum were incubated with wild type or mutated PIP region of RCC-PIP and then with PfPP1-GST. Beads were washed, suspended in Laemmli buffer, and eluates were separated by SDS–PAGE and transferred onto nitrocellulose membrane. Immunoblot analysis was performed with the anti-PfRCC-PIP antiserum (upper panel) and with anti-GST antibody (lower panel) to detect PfPP1. Lane 2 shows the interaction of PfPP1 with the wild type PIP region of PfRCC-PIP and lane 4 shows the absence of interaction with the ^980^KSASA^984^-mutated protein. As controls, an input of PfPP1-GST (lane 5), the wild type PIP region of PfRCC-PIP (lane 6) or ^980^KSASA^984^-mutated (lane 7) were used.

Next, to validate the interaction between PP1 and PfRCC-PIP, a recombinant protein corresponding to the region identified in Y2H screening was produced. This fragment corresponds to AA 863–1108 of PfRCC-PIP and is designated hereafter as the PIP region of PfRCC-PIP (Figure 1A). In order to examine whether the RCC-PIP RVXF motif is a random or a genuine binding motif, two experimental approaches were carried out using: (1) the PIP region of PfRCC-PIP in which the potential RVXF binding motif ^980^KSVSF^984^ was mutated to ^980^KSASA^984^ and (2) a PfPP1 version where the conserved RVXF binding channel involving the amino acids F255 and F256, known as major contributors in the interaction from the PP1 side (Wu and Tatchell, 2001; Hurley et al., 2007) were replaced by Ala. The capacity of interaction of the wild type and mutated constructions was assessed using the Y2H system. Mating assays of yeast transformed with different constructs are shown in Figure 3A. The growth on Double dropout medium (Ddo) demonstrated the presence of the two vectors in all mutated yeasts, and western blot analysis of yeast extracts prepared from diploids showed the expression of tagged wild type or mutated PfPP1 and wild type or mutated PIP region of RCC-PIP (Figures 3B–D). Immunoblots using extracts from respective control yeast transformed with empty or laminin vectors did not show any specific band (Figure 3B and data not shown). When mated yeasts were plated on Tdo (Triple dropout, low stringency selection) or Qdo (Quadruple dropout, high stringency selection) medium, the diploid strain containing wild type PfPP1 and wild type PIP region of RCC-PIP was able to grow, confirming the interaction between these proteins. However, the use of the mutated RVXF binding motif of RCC-PIP or the mutated RVXF binding channel of PfPP1, along with wild type PP1 or wild type RCC-PIP, respectively, did not show any yeast growth. This confirms the specificity of the interaction and suggests the implication of the RVXF motif. Side by side mating assays with constructs and negative vector controls or empty vectors did not show any yeast growth (Figure 3A).

To clarify the contribution of the RVXF binding motif, the interaction between PfPP1 and the PIP region of RCC-PIP was further investigated by immunoprecipitation experiments. For this purpose, the wild type and mutated PIP region of RCC-PIP, and GST-tagged PfPP1 were produced as recombinant proteins (Supplementary Figure 3A). An antiserum was also raised against the PIP region of PfRCC-PIP and shown to recognize both wild type and ^980^KSASA^984^-mutated proteins (Supplementary Figure 3B). Results from immunoprecipitation experiments followed by immunoblot with anti-GST antibodies indicated that GST-PfPP1 was able to efficiently bind the PIP region of PfRCC-PIP (Figure 3E, lane 2). The specificity of the interaction was validated by the absence of PfPP1 binding when a pre-immune serum was used in the immunoprecipitation (Figure 3E, lane 1). When ^980^KSASA^984^-mutated PfRCC-PIP was used in immunoprecipitation experiments, PfPP1-GST was not detected in the eluate (Figure 3E, lane 4). These results confirm the Y2H data and show that the RVXF motif of PfRCC-PIP is indispensable to this PIP region for PfPP1 binding.

### Detection of the Complex RCC-PIP/PP1 in Blood Stage Parasites

**FIGURE 4:**
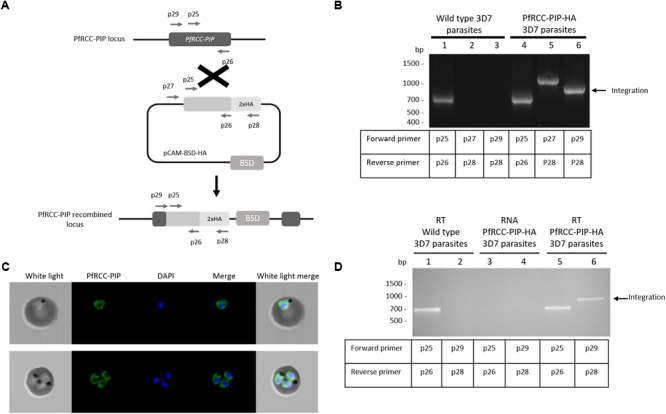
Tagging of endogenous RCC-PIP in *P. falciparum* and localization studies. **(A)** Knock-in strategy using the vector pCAM-BSD-HA that allows the insertion of an HA epitope at the C-terminus of PfRCC-PIP with a single homologous recombination. The pCAM-BSD construct, the blasticidin-resistance cassette (BSD), the location of the primers used for PCR analysis and the locus resulting from integration are shown. **(B)** Diagnostic PCR analysis of pCAM-BSD-HA-PfRCC-PIP-transfected Pf3D7 cultures; lanes 1 to 3 correspond to DNA extracted from wild type Pf3D7 parasites and lanes 4 to 6 to DNA extracted from transfected parasites. Lanes 1 and 4 represent the detection of the wild type locus (PCR with p25-p26); lanes 2 and 5 represent the detection of the construct (PCR with p27-p28); and lanes 3 and 6 correspond to the integration at the 5′ end of the insert (PCR with p29-p28). The presence of a PCR product in lane 6 (arrow) confirms the integration of an HA-tagged *PfRCC-PIP* gene in the locus. **(C)** Cellular distribution of PfRCC-PIP-HA analyzed by immunofluorescence with anti-HA antibodies. The PfRCC-PIP protein appears in green and the nucleus is stained in blue by DAPI. Upper and lower panels show one erythrocyte infected by one trophozoite and 3 trophozoites, respectively. The fluorescent signal is detected at the periphery of the nucleus. **(D)** PCR on reverse transcribed RNA from wild type (lanes 1 and 2) or knock-in PfRCC-PIP-HA parasites (lanes 5 and 6). Non-reverse transcribed RNA from PfRCC-PIP-HA parasites was used as negative control (lanes 3 and 4). PCR were performed with primers p29 and p28 to detect the integration (lanes 2, 4, and 6), and p25 and p26 for the control (lanes 1, 3, and 5). The absence of any band in lane 3 indicates the non-contamination of RNA by genomic DNA, and the presence of a fragment at the expected size in lane 6 (arrow) confirms the presence of HA-tagged PfRCC-PIP transcripts in PfRCC-PIP-HA parasites.

**FIGURE 5:**
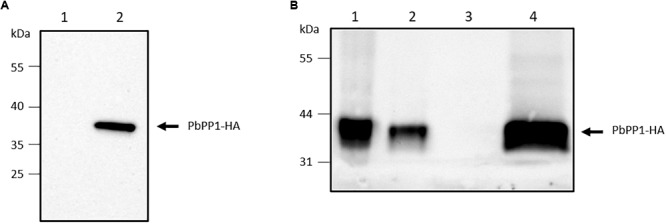
Expression of PbPP1-HA in *P. berghei*. **(A)** The expression of PbPP1-HA (lane 2) was checked in transfected *P. berghei* parasites by western blot analysis with an anti-HA antibody, and parental parasites were used as control (lane 1). **(B)** The solubility of PbPP1-HA in transfected parasites was assessed by immunoprecipitation from the soluble fraction of these parasites. Lanes 1 and 2, correspond to insoluble and soluble fractions of PbPP1-HA parasites, respectively. Lanes 3 and 4 correspond to the immunoprecipitation of the soluble fraction from parental and PbPP1-HA parasites, respectively, using anti-HA beads. The membrane was probed with anti-HA antibodies.

The data reported above clearly evidenced that the PIP region of PfRCC-PIP interacts *in vitro* with PfPP1. We next investigated whether the PP1/RCC-PIP complex could be detected in the parasite. To this end, attempts were undertaken to generate *P. falciparum* and *P. berghei* lines expressing an HA-tagged RCC-PIP using, respectively, single and double homologous recombination into the endogenous gene locus. In the case of *P. falciparum*, we introduced a targeted modification of the PfRCC-PIP locus allowing the integration of an HA-tag at the 3′ end of the PfRCC-PIP coding region (Figure 4A). Genotype analysis of viable blood stage parasites demonstrated the correct integration of the HA tag (Figure 4B). Further, using an anti-HA antibody, immunofluorescence analysis showed a distinct staining in the cytoplasm of *P. falciparum* parasites in a perinuclear zone, while no staining is observed in the nucleus of parasites (Figure 4C). Although, RT-PCR confirmed the presence of transcripts (Figure 4D), immunoblots on whole parasite extracts or on eluates after an immunoprecipitation of soluble extracts did not allow the detection of specific protein bands. Regarding *P. berghei*, attempts to generate correct constructs (PCR or optimized synthetic gene) with a GFP tag at the 3′ end of the PbRCC-PIP gene (PBANKA_0820800) were unsuccessful, likely due to stretches of Adenine and Thymine (>83% AT). Moreover, HA tagging PbRCC-PIP at its 5′ end did not allow the detection of an integration of the tag (not shown), although viable parasites were obtained. To overcome these issues and based on the *in vitro* data showing that the PIP region of RCC-PIP was able to interact with PP1, a *P. berghei* line expressing an HA-tagged PP1 (PBANKA_1028300) was generated (Supplementary Figure 4A). Genotype analysis by specific PCR of these parasites, compared to parental parasites, showed an integration of the HA epitope at the 5′ end of the PbPP1 gene (Supplementary Figure 4B). The expression of HA-tagged PbPP1 was detected in the soluble fraction of parasites (Figure 5A, lane 2), as well as in a fraction immunoprecipitated using anti-HA agarose beads (Figure 5B, lane 4). However, western blot analysis of this fraction, using the anti-PfRCC-PIP antiserum did not allow the detection of a specific band at the expected size. This could be due to the high MW of the protein (predicted MW 292 kDa) which may be inefficiently transferred onto nitrocellulose, to a weak expression of the protein or to low affinity of produced antibodies for the *P. berghei* native protein. This prompted us to perform immunoprecipitation/Mass spectrometry (IP/MS) experiments. *P. berghei* extracts from HA-PP1 expressing parasites were immunoprecipitated using anti-HA agarose beads. Control IP was performed using extracts prepared from the untagged parental *P. berghei* strain. In two independent IP/MS experiments, analysis of IP fraction of HA-PbPP1 expressing parasites revealed the highest enrichment of the bait PP1, with the presence of 25 and 19 peptides in experiments 1 and 2, respectively (Table 1). Although 4 peptides of PbPP1 were identified in the control in experiment 1, very low assigned spectra were detected (5 vs. 288 spectra in HA-PbPP1 fraction). Importantly, in the top ranking proteins identified, the two following proteins correspond to PbLRR1 and PbI2. Our earlier studies have shown that their orthologs in Pf (PfLRR1 and PfI2) were able to interact with PP1 *in vitro* (Daher et al., 2006; Freville et al., 2013). These results not only reflect that these interactions could take place in parasites, but also support the quality and robustness of IP/MS approach. In the case of PbRCC-PIP, it was detected in the IP fraction from the HA-PbPP1 strain but not from the parental strain, with 24 and 14 peptides identified in experiments 1 and 2, respectively (total number of spectrum 35 and 26, respectively), and 10 peptides common to both experiments (Table 1 and Supplementary Table 2). These data, together with *in vitro* experiments, strongly indicate a direct and physical interaction of RCC-PIP with PP1.

### Functional Activity of the PIP Region of PfRCC-PIP in *Xenopus laevis* Oocytes

Having demonstrated that the PIP region of PfRCC-PIP interacts with PfPP1, we next investigated its potential capacity to regulate PfPP1 activity. This was first addressed in an *in vitro* assay in which we measured the capacity of the PIP region of PfRCC-PIP to modulate the dephosphorylation of the *p*-Nitrophenyl Phosphate (pNPP) non-specific substrate by recombinant PfPP1. Increasing concentrations of recombinant PIP region of PfRCC-PIP did not show any significant effect on PfPP1 activity (data not shown). We then turned our attention to the *Xenopus* oocyte model in which we have previously shown that several phosphatase partners could regulate cell-cycle progression from G2 to M, assessed by the appearance of GVBD (Daher et al., 2007; Freville et al., 2013; Vandomme et al., 2014). Interestingly, PP1 is a highly conserved enzyme in different species. Sequence comparative analysis revealed 84% amino acid identity between *Plasmodium* PP1 and *Xenopus* PP1 (Supplementary Figure 5). This allows to explore the PP1 interaction network in *Xenopus* oocytes. In this model, the preinjection into oocytes of an inhibitor of phosphatases will trigger the G2/M transition, while a phosphatase activator will lead to an inhibition of the progesterone-induced maturation.

**FIGURE 6:**
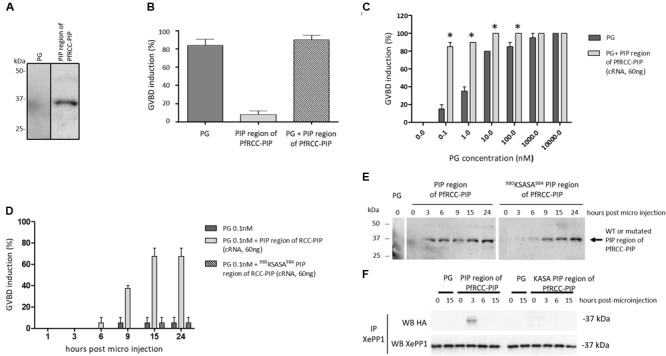
Expression of the PIP region of PfRCC-PIP in *Xenopus laevis* oocytes and role on the induction of Germinal Vesicle BreakDown. **(A)** Production of the PIP region of PfRCC-PIP in *Xenopus* oocytes. 15 h after micro-injection of the PIP region of PfRCC-PIP mRNA in *Xenopus* oocytes, the production of the corresponding protein was checked by western blot probed with an anti-HA antibody. A native oocyte lysate was used as negative control in lane PG. **(B–D)** Role of the PIP region of PfRCC-PIP on the induction of Germinal Vesicle BreakDown (GVBD). *Xenopus* oocytes were micro-injected with mRNA of PIP region PfRCC-PIP and were then incubated with or without progesterone (PG) at different concentrations, and GVBD was observed 16 h post injection **(B,C)** or every 3 h post injection **(D)**. **(B)** Percentage of GVBD induced by PG (10 μM), by the PIP region of PfRCC-PIP, with or without PG incubation. **(C)** Percentage of GVBD induced by the PIP region of RCC-PIP in presence of increasing concentrations of PG (0.1 nM to 10 μM). In parallel, oocytes without micro-injection of the PIP region of RCC-PIP were used as control and incubated in the same concentrations of PG. Results are expressed as GVBD percentage (+/–SEM, *n* = 2), and the Mann-Whitney test was used to evaluate the significance with control oocytes (PG only). ^∗^*P* < 0.05. **(D)** Kinetic analysis of GVBD induced by wild type vs. mutated PIP region of RCC-PIP in presence of 0.1 nM of PG. **(E)** Kinetic analysis of protein expression in oocytes lysates after microinjection of mRNA encoding wild type vs. mutated PIP region of PfRCC-PIP. The membrane was probed with anti-HA mAb. **(F)** Interaction between XePP1 and the PIP region of PfRCC-PIP analyzed by co-immunoprecipitation. The XePP1-PfRCC-PIP complex was immunoprecipitated (IP) with anti-XePP1 antibodies from microinjected *Xenopus* oocytes, separated by SDS–PAGE and transferred to a nitrocellulose membrane. Western blot (WB) analysis was performed with anti-XePP1 antibodies (lower panel) or anti-HA antibodies (recognizing PfRCC-PIP) (upper panel). A band is observed in upper panel in lysates from oocytes microinjected with wild type PIP region of PfRCC-PIP 3 h after microinjection, indicating an interaction with XePP1.

We first checked that the microinjection of the cRNA encoding the PIP region of PfRCC-PIP induced the expression of the corresponding protein in oocyte lysates by western blot (Figure 6A). At the functional level, the microinjection of this cRNA alone did not induce the maturation of *Xenopus* oocyte GVBD and was unable to block the GVBD induced by 10 μM progesterone (Figure 6B). On the contrary, in an additional series of experiments in which the GVBD was induced in the presence of lower concentrations of progesterone, we observed an increase of the percentage of GVBD in oocytes microinjected with the cRNA of PfRCC-PIP. This prompted us to further investigate the effect of the microinjection of the PIP region of PfRCC-PIP on the GVBD induced by sub-optimal concentrations of progesterone. As shown in Figure 6C, the microinjection of the PIP region of PfRCC-PIP in oocytes incubated in presence of concentrations of progesterone higher than 1 μM did not affect the induction of GVBD, confirming the results shown in Figure 6B. However, when progesterone was added to the oocyte medium at lower concentrations, we observed a significant increase of the induction of GVBD in the presence of the PIP region of PfRCC-PIP (Figure 6C). To explore the contribution of the RVXF motif, we performed a kinetic assay in which GVBD was followed during 24 h after microinjection of wild type vs. ^980^KSASA^984^-mutated PIP region of PfRCC-PIP, in the presence of 0.1 nM progesterone. The GVBD appeared as soon as 9 h post microinjection when the wild type construct was used (Figure 6D). However, when the cRNA of ^980^KSASA^984^-mutated PIP region of RCC-PIP was micro-injected no GVBD was observed (Figure 6D), although the production of the corresponding recombinant protein was detected (Figure 6E). The co-immunoprecipitation/immunoblot assays performed on oocyte lysates revealed an interaction between *Xenopus* PP1 and the PIP region of PfRCC-PIP 3 h post microinjection (Figure 6F). However, no interaction was detected when the mutated PIP region was injected. Taken together, these observations suggest that the induction of GVBD by the PIP region of PfRCC-PIP in presence of low concentrations of progesterone is related to a functional interaction of the PIP region of PfRCC-PIP with *Xenopus* PP1.

### Interaction of the RCC Region of PfRCC-PIP With PfCDPK7

Given the known ability of RCC domains to interact with proteins, a Pf cDNA Y2H library was screened using the RCC region of PfRCC-PIP (Nt portion spanning 399 amino acids and containing the 2 RCC domains, Figure 1A) as bait. Two clones in frame with GAL4-AD were obtained.

**FIGURE 7:**
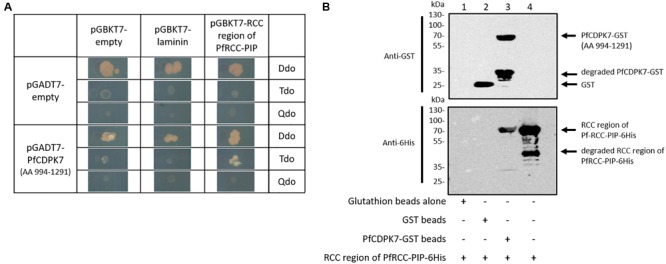
Interaction between RCC region of RCC-PIP and PfCDPK7. **(A)** Interaction between the RCC region of RCC-PIP and PfCDPK7 in yeast two-hybrid approach. The RCC region of PfRCC-PIP (AA 1–399) was cloned in pGBKT7 vector and the construct was inserted in mat α yeast (Y187). pGADT7 transformed with a fragment of PfCDPK7 (AA 994–1291) isolated from Y2H screening as a potential partner of PfRCC-PIP, was inserted in mat a (Y2HGold) yeast. After mating, diploids were checked on Ddo medium, interactions and strong interactions were examined on Tdo and Qdo medium, respectively. pGADT7 empty, pGBKT7 empty and pGBKT7-laminin were inserted in corresponding yeast strains and used as controls. **(B)** Direct interaction between RCC region of PfRCC-PIP and PfCDPK7 (AA 994–1291) assessed by GST pulldown assay. Glutathione beads alone (lane 1) or coupled with GST (lane 2), or with PfCDPK7-GST (lane 3) were incubated with the His-tagged recombinant RCC region of PfRCC-PIP. After washes, proteins bound to the beads were separated by SDS–PAGE and blotted onto nitrocellulose. Immunoblot analysis was performed with anti-GST mAb (upper panel) recognizing GST and PfCDPK7-GST proteins and anti-His mAb antibodies (lower panel) recognizing the recombinant RCC region of PfRCC-PIP. As a control, an input of His-tagged RCC region of PfRCC-PIP was used (lane 4).

Further confirmation experiments in a second screen with controls including empty and laminin vectors and the two selected clones showed that only one clone exhibited specific binding (Figure 7A). The second clone, inducing yeast growth with control constructs that could be due to spurious GAL4 promoter-binding prey proteins was excluded. Blast analysis of the sequence of the positive clone, encoding 297 amino acids, showed that it corresponded to an atypical kinase of *P. falciparum*, the PfCDPK7 (PF3D7_1123100). To validate our data by an independent biochemical approach, the PfCDPK7 binding region (AA 994–1291) and the RCC region of PfRCC-PIP (AA 1–399) were produced as recombinant proteins tagged with GST and 6 × His, respectively and examined for their capacity to interact. As shown in Figure 7B, the binding region of PfCDPK7 fused to GST was able to pull down the RCC region-containing protein (Figure 7B, lane 3), whereas GST alone did not (Figure 7B, lane 2). This confirms the interaction detected by the Y2H approach and indicates a direct binding between the two proteins.

## Discussion

Key steps of *Plasmodium* life cycle are under the control of the balance between phosphorylation and dephosphorylation. Protein kinases and phosphatases, which subtly regulate these reactions, are thus critical enzymes for parasite survival. The spatio-temporal control of their activity is achieved through their interaction with a variety of proteins. We describe here a new *Plasmodium* phosphatase- and kinase-interacting protein that we named RCC-PIP due to its RCC-1 homolog- and PP1-interacting domains. First, in an attempt to explore the role of RCC-PIP in *Plasmodium* life cycle, we tried to disrupt its gene. In our study, no viable disrupted *P. falciparum* or *P. berghei* parasites were obtained despite several attempts, suggesting an essential role for RCC-PIP in blood stage parasites. Very recently, *P. falciparum* mutants have been generated via random PiggyBac transposon mutagenesis (Zhang et al., 2018), which suggested that PfRCC-PIP was a dispensable gene when the Mutagenesis Index Score (MIS) was used as readout, while the Mutagenesis Fitness Score (MFS) value indicated that it seems to be essential. These data should be interpreted cautiously until the function is assessed by gene inducible knock down approach. In addition, earlier study using high through put knock out screening in *P. berghei* did not allow the detection of viable disrupted RCC-PIP parasites (Bushell et al., 2017).

PfRCC-PIP had been previously identified as one of the major interactor of PfPP1, shown as an essential phosphatase for blood stage parasites, in yeast two-hybrid screening (Hollin et al., 2016). This was further supported by the presence of an extended RVXF binding consensus sequence (Hollin et al., 2016). In the present study, we have confirmed the interaction of the PIP region of PfRCC-PIP with PfPP1 using immunoprecipitation experiments, indicating a direct interaction between the two proteins. Furthermore, we have shown by two independent experimental approaches that the putative RVXF motif present in the PIP region is functional since its mutation abolished the interaction with PfPP1. This was further validated by the use of a PfPP1 mutant in which the amino acids F255 and F256, known to be involved in the RVXF binding (Wu and Tatchell, 2001; Hurley et al., 2007), were mutated.

Since PP1 interactors may be regulators, substrates and/or connecting/chaperone proteins, we investigated whether the PIP region of PfRCC-PIP may be functional by regulating PfPP1 activity. *In vitro* experiments using pNPP as a small non-physiological substrate, did not show any modulation of PP1 activity by RCC-PIP (data not shown). To further investigate PfRCC-PIP functional role, we took advantage of the *Xenopus* model where oocytes are physiologically arrested in G2/M prophase. In this model, we showed that the PIP region of RCC-PIP interacts with XePP1, which is highly conserved with PfPP1, in cellular context. This interaction involves the RVXF motif of the PIP region of PfRCC-PIP, since the ^980^KSASA^984^ mutant did not show any interaction with XePP1. In a first series of experiments, the microinjection of the cRNA of the PIP region of PfRCC-PIP alone, or in the presence of high concentrations of progesterone, did not allow to either induce GVBD or modulate the progesterone-induced GVBD, respectively. This suggests that PfRCC-PIP may be neither an inhibitor, nor an activator of XePP1. However, when suboptimal doses of progesterone were used, we observed an increase in the induction of GVBD in the presence of the PIP region of RCC-PIP. This effect was also dependent on the RVXF motif of PfRCC-PIP. These observations indicate that the microinjection of the PIP region of PfRCC-PIP and the presence of progesterone are clearly two synergized events to induce GVBD. Of note, the progesterone-induced GVBD in *Xenopus* oocytes is characterized by feedback loops of the downstream MAPK and MPF (cdc2/cyclinB) signaling cascades that are capable to potentiate upstream events (Ferrell and Machleder, 1998; Russo et al., 2009). GVBD induced by low concentrations of progesterone can be potentiated by extracellular addition of insulin growth factor at a concentration that had no apparent effect on GVBD on its own or by intracellular injections of modulators that directly target kinases and/or phosphatases involved in these feedback events. Another characteristic of oocyte GVBD is that the amplified signal only needs one initial stimulating event to start and does not require a continuous stimulation or a continuous interaction between partners (Le Goascogne et al., 1984; Schmitt and Nebreda, 2002). The disruption of the interaction between *Xenopus* PP1 and the PIP region of RCC-PIP could be due to a competition with endogenous PP1 partners expressed by *Xenopus* oocytes. This early binding suggests that the interaction of the two partners is necessary to trigger an initial step in the signaling cascades necessary for GVBD that is later increased, by the action of a feedback loop. PP1 activity is necessary in the process of progesterone-induced oocyte GVBD acting on the MPF (cdc2/cyclinB) signaling cascade where it modulates a series of specific phosphorylation sites (Huchon et al., 1981; Margolis et al., 2006). We can suppose that the action of the PIP region of PfRCC-PIP is sufficient to strengthen the action of PP1 on the MPF cascades and that this event is potentiated by the action of suboptimal doses of progesterone on the same MPF and on the other typical signaling cascades triggered by progesterone [such as the MAPK cascade and AMPc (Ferrell and Machleder, 1998)].

While these results confirm the interaction of the PIP region of PfRCC-PIP with PP1, and indicate a functional role of this region which can be related to the interaction with PP1, we have to keep in mind that they have been observed using a fragment of the protein. The total native protein, which is very large size (3381 AA, predicted MW 396 kDa), may behave differently. We thus investigated the interaction of RCC-PIP and PP1 in the parasite. For this purpose, knock-in *P. falciparum* parasites expressing HA-tagged PfRCC-PIP were generated. These lines allowed the observation of the cytoplasmic localization of PfRCC-PIP in blood stage parasites. However, we were unable to detect the tagged protein in parasites extracts by western blot analysis. This could be linked to technical limitations due to the large size of the protein. The HA tag may also be not accessible to the antibodies used in these experiments. It is of note that neither the native nor the tagged RCC-PIP were detected in western blot analysis using the antiserum directed against the PIP region of RCC-PIP. To overcome the difficulty, we generated a *P. berghei* HA-PbPP1-expressing line. Using these parasites in immunoprecipitation assays, HA-PbPP1 was detected in soluble extracts, but PbRCC-PIP was still undetectable. We then performed Mass Spectrometry on the immunoprecipitated fraction. In two independent experiments IP/MS showed the presence of peptides specific for PbRCC-PIP, demonstrating the presence of PP1/RCC-PIP complexes in *P. berghei*. Taken together, these results confirm that RCC-PIP is a real interactor of PP1 in blood stage parasites, and *in vitro* data shows that it may interact directly via its RVXF motif contained in the PIP region of the protein.

Further analysis of PfRCC-PIP amino acid sequence revealed that, beside an RVXF motif, it exhibits two Regulator of Chromosome Condensation (RCC1) consensus motifs and one RCC1 signature 2 at the Nt moiety of the protein. These motifs were initially described in human RCC1 protein, in which seven of these motifs are observed and adopt a seven-bladed beta-propeller fold (Ohtsubo et al., 1987; Renault et al., 1998). Each of the blades is composed of a 4-stranded antiparallel beta-sheet (Renault et al., 1998) and the overall structure of the protein allows it to interact both with Ran-GTP, as well as with nucleosomes and histones H2A and H2B (Bischoff and Ponstingl, 1991; Nemergut et al., 2001). In PfRCC-PIP, we detected only 2 RCC1 motifs. To further characterize these motifs, we built a 3D model of its RCC region using human RCC1 as template. The predicted structure suggests that the RCC motifs of PfRCC-PIP may interact with proteins and/or DNA. Since the cellular distribution of HA-tagged PfRCC-PIP showed a cytoplasmic (perinuclear) localization of the protein, we explored the possible interaction of its RCC region with proteins. This allowed the identification of a fragment of PfCDPK7, a calcium-dependent protein kinase (CDPK), as an interactor of PfRCC-PIP. CDPKs are characterized by a kinase domain fused to a calmodulin-like domain and are major actors in calcium signaling in plants and apicomplexan parasites (Harper and Harmon, 2005). *P. falciparum* possesses seven annotated CDPKs characterized by a carboxyl-terminal tail containing two to four calcium-binding EF-hand domains (Billker et al., 2009). PfCDPK7 is a large size protein kinase (265 kDa, 2265 AA) that harbors two EF-hand domains in its Nt moiety, and a pleckstrin-homology domain (PH) just upstream of its serine/threonine kinase domain found at the Ct end of the protein. PfCDPK7 has been shown to interact with PI(4,5)P2 via its PH domain, and to play an important role during the asexual stages of the parasite, with a marked reduction of maturation of rings to trophozoite stages in deficient parasites (Kumar et al., 2014). In the recent study using random PiggyBac transposon mutagenesis, PfCDPK7 appears to be essential (Zhang et al., 2018). Moreover, the *P. berghei* homolog of PfCDPK7 (PBANKA_0925200), has been shown to be essential in two independent studies (Tewari et al., 2010; Schwach et al., 2015). The fragment of PfCDPK7 isolated in Y2H screening (AA 994–1291) is located away from the characteristic domains of the protein. We further produced this fragment as a recombinant protein fused to GST, and included it in a GST pull down assay. This allowed us to confirm that the RCC motifs of PfRCC-PIP directly interact with PfCDPK7. Further investigations are still required to demonstrate this interaction and its functional impact in the parasite.

In conclusion, our work indicates that RCC-PIP interacts both with a phosphatase (PP1), via its RVXF motif, and a kinase (CDPK7), via a region containing the RCC motifs. Such bifunctional proteins able to interact with kinases and phosphatases have already been described in mammalian cells. These include A-Kinase Anchoring Proteins (AKAP), which are a genetically diverse but functionally related family of proteins that interact with Protein kinase A (PKA) and also with other signaling enzymes among which are phosphatases (Wong and Scott, 2004). D-AKAP1 (AKAP149), AKAP220, and WAVE1 have been shown to be AKAPs that interact with PP1 via their RVXF motif (Schillace and Scott, 1999; Steen et al., 2000; Danial et al., 2003). Some of these proteins contribute to the recruitment of PKA and PP1 to specific subcellular environments, and thus to regulate the phosphorylation events (Steen et al., 2000; Danial et al., 2003). Whether the role of PfRCC-PIP in the parasite may be also to transport PP1 and/or PfCDPK7 to a specific localization or to a specific substrate should be investigated. Also, the analysis of the potential cross regulation of the phosphorylation of these three partners could help in elucidating the role of this PP1- and CDPK7-anchoring protein in *Plasmodium*.

## Author Contributions

CP and JK designed the study. AL, CW, GT, TH, EA, AM, KC, and J-MS performed the experiments. AL, JK, and CP wrote the paper. All authors analyzed the data, read, contributed feedback to, and approved the final manuscript.

## Conflict of Interest Statement

The authors declare that the research was conducted in the absence of any commercial or financial relationships that could be construed as a potential conflict of interest.

## Footnotes

1 https://modbase.compbio.ucsf.edu/scgi/modweb.cgi

## Abbreviations

CDPK: calcium-dependent protein kinase
GVBD: germinal vesicle breakdown
PP1: protein phosphatase type-1
RCC-PIP: regulator of chromosome condensation-PP1-interacting protein
Y2H: yeast two hybrid
