# Supplementary material for: Characterization of a Protein Phosphatase Type-1 and a Kinase Anchoring Protein in Plasmodium falciparum
Source: Front Microbiol. 2018 Oct 31;9:2617. doi: 10.3389/fmicb.2018.02617 (PMC6220109; doi:10.3389/fmicb.2018.02617)
Supplement: Supplementary file 2 [file Data_Sheet_1.PDF]

Supplementary Table 2. PbRCC-PIP peptides identified in IP fractions from HA-PbPP1 parasites

Experiment 1 [a, b]	Experiment 2 [a, b]
**10-INLFIINDVNNNDcK-24**	**10-INLFIINDVNNNDcK-24**
	29-TESTLFVDK-37
**38-QEADNININEK-48**	**38-QEADNININEK-48**
**72-SIIAFLSQIGK-82**	**72-SIIAFLSQIGK-82**
	97-NVPYLLIDNILK-108
111-TIHDVSSGSNHIAFVSK-127	
**226-TLLHPHLIDTNNIAFK-241**	**226-TLLHPHLIDTNNIAFK-241**
303-TIDGDIYIWGGTYGNKPNIIK-323	
324-NKFDSLHINNNYIIGLcSK-342	
349-NINTSSHGYYINNLK-363	
**364-INLISSYDNLIIGVDNFLQSDNVVDAK-390**	**364-INLISSYDNLIIGVDNFLQSDNVVDAK-390**
**398-NSNSLLETNSESVSNNGK-415**	**398-NSNSLLETNSESVSNNGK-415**
547-NNLNNNKDDVcDLK-560	
561-NYNENGEVHNFKDQINENK-579	
**621-TYGQTPNSIPDNPIIDNSFSVK-642**	**621-TYGQTPNSIPDNPIIDNSFSVK-642**
	643-AFEIEGKNESK-653
657-INGLNNSDLINFSFSEYNEIQTK-679	
**680-ESIPFNFNPIcK-691**	**680-ESIPFNFNPIcK-691**
692-IPNDSNYIDNINNNDDSIFEmDSNYNDKK-720	
**727-LNmENENIDNIK-738**	**727-LNmENENIDNIK-738**
765-YYDEEDDFFSTNFYGLSESDYLDAK-789	
**790-NIIIINNDENNPNLK-804**	**790-NIIIINNDENNPNLK-804**
	810-TPSTTDIRK-817
868-NDQLNESNIMDFEANNIDNTcR-889	
903-VESDDPNEVDVYDINK-918	
903-VESDDPNEVDVYDINKDSEHNFK-925	
977-SSNNIHLIK-985	
1021-YYESTSSEEKNNLK-1034	

[a] Amino acid sequences and positions are indicated; common peptides in the two experiments are bolded.
[b] c: carbamidomethyl cysteine, m: oxidized methionine
