# Supplementary figures and images for: Characterization of a Protein Phosphatase Type-1 and a Kinase Anchoring Protein in Plasmodium falciparum
Source: Front Microbiol. 2018 Oct 31;9:2617. doi: 10.3389/fmicb.2018.02617 (PMC6220109; doi:10.3389/fmicb.2018.02617)

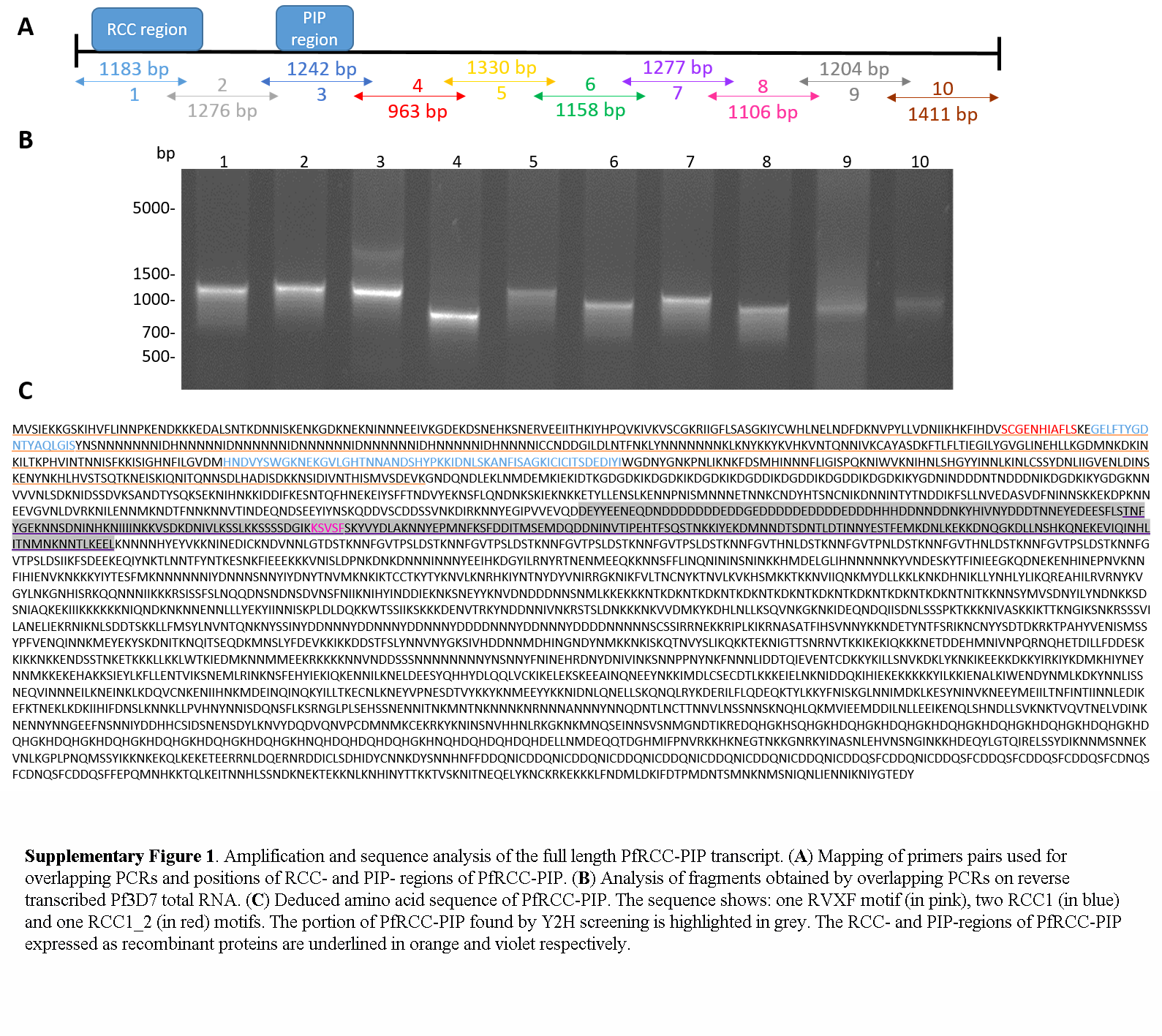

Supplement: Supplementary file 4 [file Image_1.TIF]

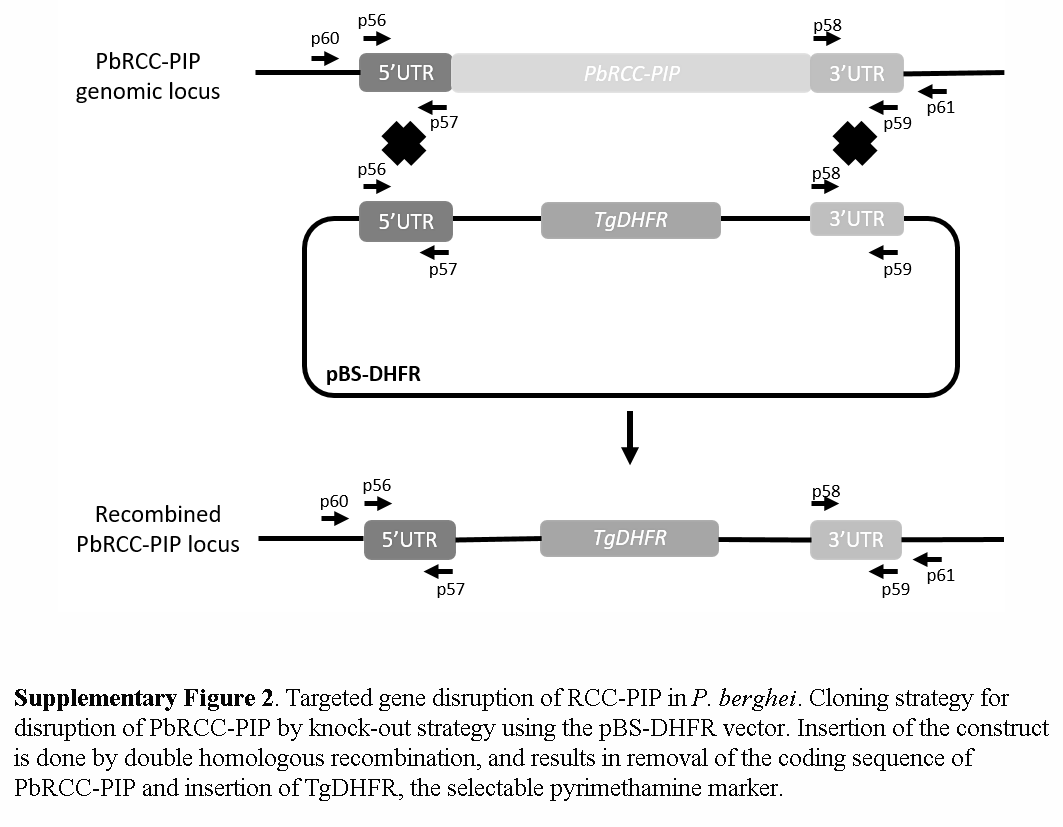

Supplement: Supplementary file 5 [file Image_2.TIF]

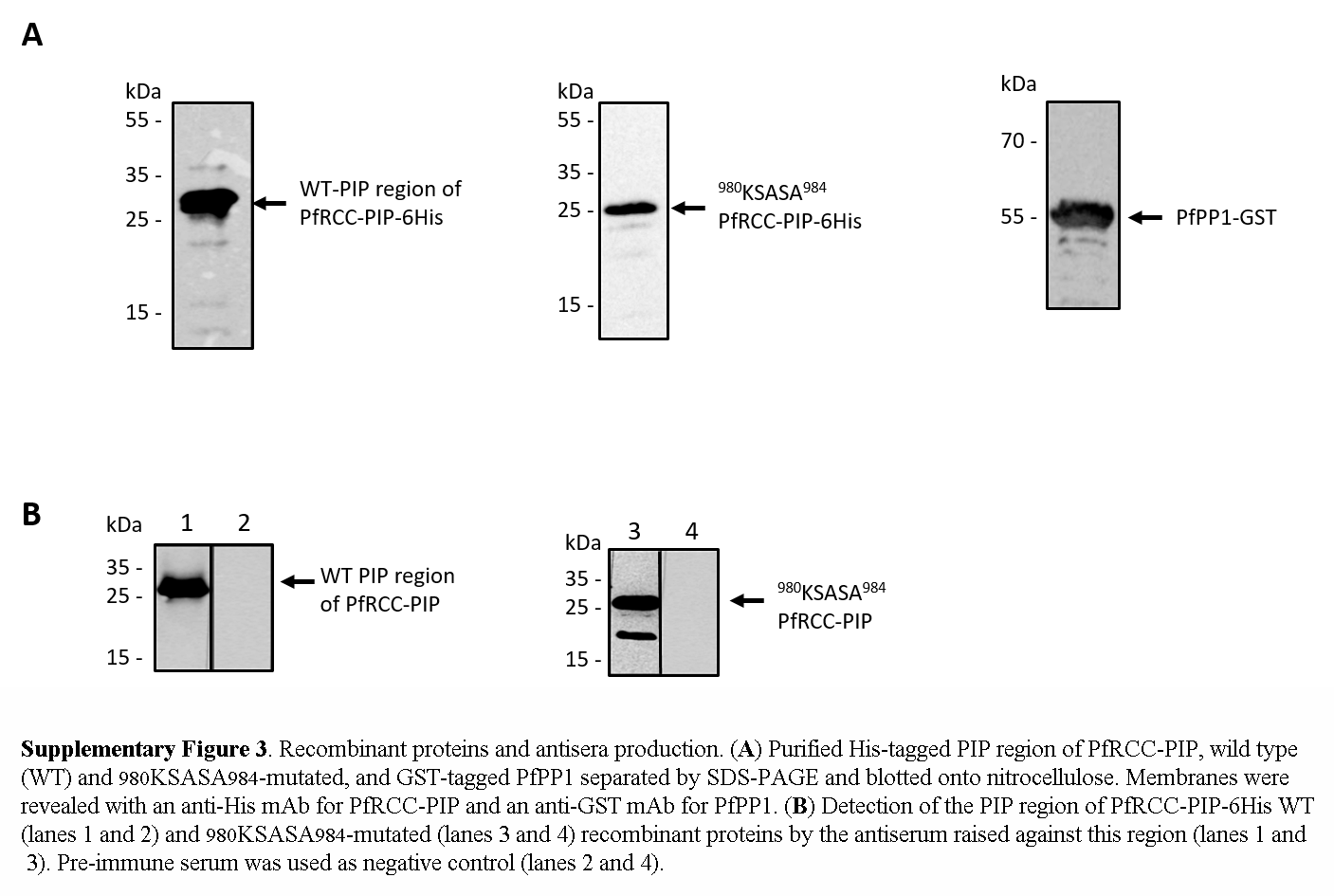

Supplement: Supplementary file 6 [file Image_3.TIF]

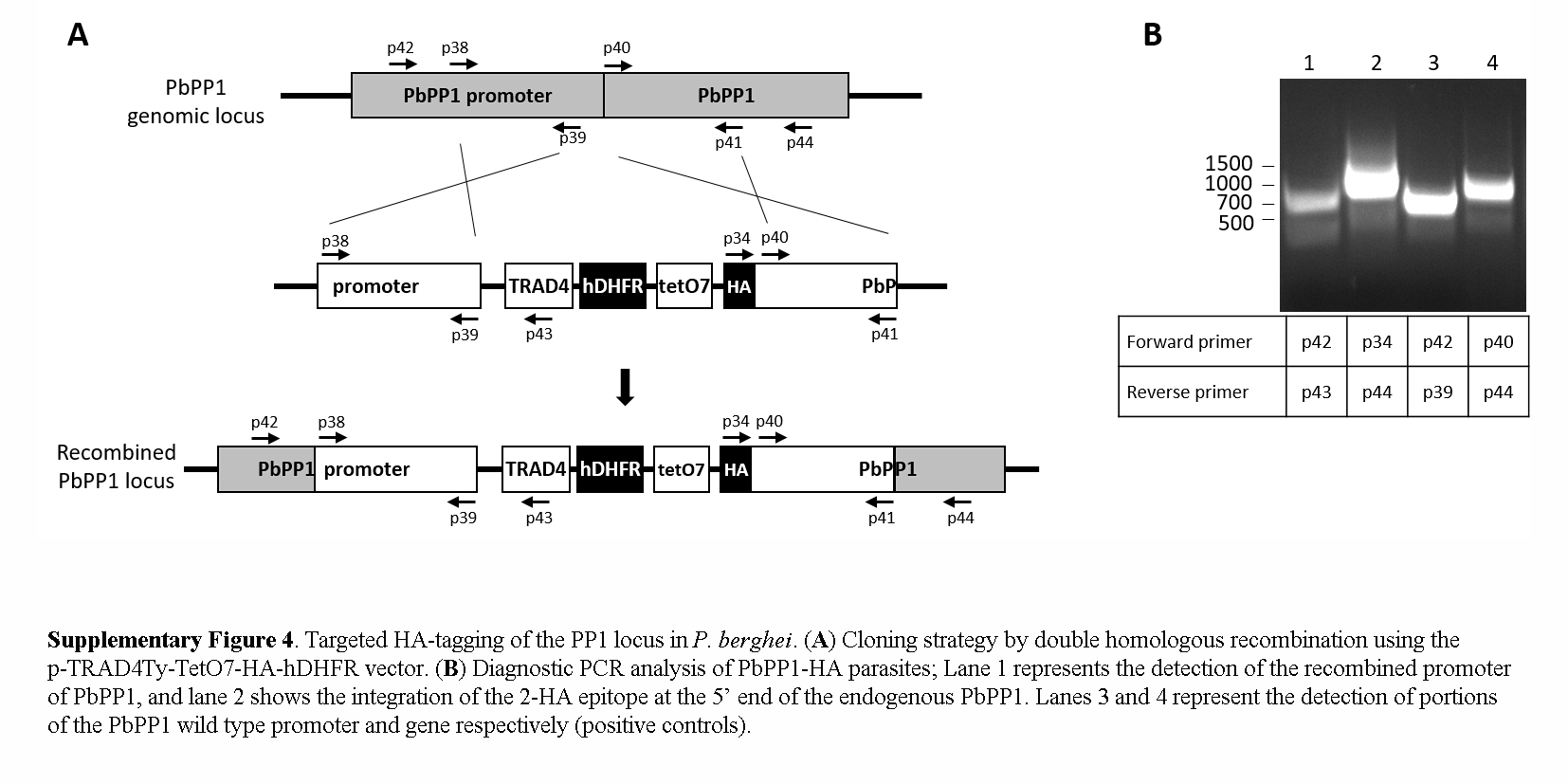

Supplement: Supplementary file 7 [file Image_4.TIF]
