# Supplementary material for: Characterization of a Protein Phosphatase Type-1 and a Kinase Anchoring Protein in Plasmodium falciparum
Source: Front Microbiol. 2018 Oct 31;9:2617. doi: 10.3389/fmicb.2018.02617 (PMC6220109; doi:10.3389/fmicb.2018.02617)
Supplement: Supplementary file 3 [file Data_Sheet_2.PDF]

```
PfPP1         --MALEIDIDNVISKLIEVRGTRPGKNVNLTENEIKILCLSSREIFLNQPILLELEAPIK
PbPP1         --MALEIDIDNVISKLIEVRGTRPGKNVNLTENEIKILCLSSREIFLNQPILLELEAPIK
XePP1         MADVDKLNIDSIIQRLLEVRGSKPGKNVQLQENEIRGLCLKSREIFLSQPILLELEAPLK
humanPP1      MSDSEKLNLDSIIGRLLEVQGSRPGKNVQLTENEIRGLCLKSREIFLSQPILLELEAPLK
                   ::::*.:* .*:**.*:.*****:* ****. ***.******.**********:*

PfPP1         ICGDIHGQFYDLLRLFEYGGFPPDANYLFLGDYVDRGKQSLETICLLLAYKIKYPENFFL
PbPP1         ICGDIHGQFYDLLRLFEYGGFPPDANYLFLGDYVDRGKQSLETICLLLAYKIKYPENFFL
XePP1         ICGDIHGQYYDLLRLFEYGGFPPESNYLFLGDYVDRGKQSLETICLLLAYKIKYPENFFL
humanPP1      ICGDIHGQYYDLLRLFEYGGFPPESNYLFLGDYVDRGKQSLETICLLLAYKIKYPENFFL
              ********:**************::***********************************

PfPP1         LRGNHECASINRIYGFYDECKRRYSVKLWKTFIDCFNCLPVAAIIDEKIFCMHGGLSPEL
PbPP1         LRGNHECASINRIYGFYDECKRRYSVKLWKTFIDCFNCLPVAAIIDEKIFCMHGGLSPEL
XePP1         LRGNHECASINRIYGFYDECKRRYNIKLWKTFTDCFNCLPIAAIVDEKIFCCHGGLSPDL
humanPP1      LRGNHECASINRIYGFYDECKRRYNIKLWKTFTDCFNCLPIAAIVDEKIFCCHGGLSPDL
              ************************.:****** *******:***:****** ******:*

PfPP1         NNMEQIRKITRPTDVPDNGLLCDLLWSDPEKEINGWGENDRGVSFTFGQDVVHNFLRKHE
PbPP1         NNMEQIRKITRPTDVPDNGLLCDLLWSDPEKEINGWGENDRGVSFTFGQDVVHNFLRKHE
XePP1         QSMEQIRRIMRPTDVPDQGLLCDLLWSDPDKDILGWGENYRGVSFTFGAEVVAKFLHKHD
humanPP1      QSMEQIRRIMRPTDVPDQGLLCDLLWSDPDKDVQGWGENDRGVSFTFGAEVVAKFLHKHD
              :.*****.* *******:***********:*:: ***** ******** :** :**.**:

PfPP1         LDLICRAHQVVEDGYEFFAKRQLVTLFSAPNYCGEFDNAGAMMSVDETLMCSFQILKPVE
PbPP1         LDLICRAHQVVEDGYEFFAKRQLVTLFSAPNYCGEFDNAGAMMSVDETLMCSFQILKPVE
XePP1         LDLICRAHQVVEDGYEFFAKRQLVTLFSAPNYCGEFDNAGAMMSVDETLMCSFQILKPAE
humanPP1      LDLICRAHQVVEDGYEFFAKRQLVTLFSAPNYCGEFDNAGAMMSVDETLMCSFQILKPAD
              **********************************************************.:

PfPP1         KKKAAN--------------------------
PbPP1         KKKITT--------------------------
XePP1         KKKPNA---------SRPVTPPRGMITKQAKK
humanPP1      KNKGKYGQFSGLNPGGRPITPPRN--SAKAKK
              *:*
```

Supplementary Figure 5. Alignment of Protein phosphatase type 1 (PP1) from P.falciparum (PfPP1, PF3D7_1414400), P. berghei (PbPP1, PBANKA_1028300), Xenopus laevis (XePP1, AAA49934.1) and human (NP_002699.1). CLUSTAL multiple sequence alignment was performed using MUSCLE (3.8) software (Edgar, R.C., 2004, MUSCLE: multiple sequence alignment with high accuracy and high throughput. Nucleic Acids Research 32(5):1792-1797). Amino acids in red are small and hydrophobic amino acids, those in blue are acidic, those in magenta are basic and in green are the other amino acids. An * (asterisk) indicates positions which have a single, fully conserved residue, a : (colon) indicates conservation between groups of strongly similar properties and a . (period) indicates conservation between groups of weakly similar properties.
